# Disrupted Functional Rich-Club Organization of the Brain Networks in Children with Attention-Deficit/Hyperactivity Disorder, a Resting-State EEG Study

**DOI:** 10.3390/brainsci11070938

**Published:** 2021-07-16

**Authors:** Maliheh Ahmadi, Kamran Kazemi, Katarzyna Kuc, Anita Cybulska-Klosowicz, Mohammad Sadegh Helfroush, Ardalan Aarabi

**Affiliations:** 1 Department of Electrical and Electronics Engineering, Shiraz University of Technology, Shiraz 7155713876, Iran; ma.ahmadi@sutech.ac.ir (M.A.); ms_helfroush@sutech.ac.ir (M.S.H.); 2 Institute of Psychology, SWPS University of Social Sciences and Humanities, 03-815 Warsaw, Poland; kkuc@swps.edu.pl; 3 Laboratory of Emotions Neurobiology, Nencki Institute of Experimental Biology, Polish Academy of Sciences, 02-093 Warsaw, Poland; a.cybulska@nencki.edu.pl; 4 Laboratory of Functional Neuroscience and Pathologies (LNFP, EA 4559), University Research Center (CURS), University Hospital, 80054 Amiens, France; 5 Faculty of Medicine, University of Picardy Jules Verne, 80036 Amiens, France

**Keywords:** EEG, cortical source imaging, eLORETA, rich-club organization, graph analysis, connectivity analysis, children, combined and inattentive ADHD

## Abstract

Growing evidence indicates that disruptions in the brain’s functional connectivity play an important role in the pathophysiology of ADHD. The present study investigates alterations in resting-state EEG source connectivity and rich-club organization in children with inattentive (ADHD_I_) and combined (ADHD_C_) ADHD compared with typically developing children (TD) under the eyes-closed condition. EEG source analysis was performed by eLORETA in different frequency bands. The lagged phase synchronization (LPS) and graph theoretical metrics were then used to examine group differences in the topological properties and rich-club organization of functional networks. Compared with the TD children, the ADHD_I_ children were characterized by a widespread significant decrease in delta and beta LPS, as well as increased theta and alpha LPS in the left frontal and right occipital regions. The ADHD_C_ children displayed significant increases in LPS in the central, temporal and posterior areas. Both ADHD groups showed small-worldness properties with significant increases and decreases in the network degree in the θ and β bands, respectively. Both subtypes also displayed reduced levels of network segregation. Group differences in rich-club distribution were found in the central and posterior areas. Our findings suggest that resting-state EEG source connectivity analysis can better characterize alterations in the rich-club organization of functional brain networks in ADHD patients.

## 1. Introduction

Attention deficit hyperactivity disorder (ADHD), as one of the most common neurodevelopmental disorders, affects 2.2–17.8% of all school-aged children and adolescents [1,2]. ADHD is categorized into three subtypes: a rarely identified hyperactive-impulsive subtype, an inattentive subtype (ADHD_I_) and the most common combined subtype (ADHD_C_), characterized by both inattention and hyperactivity (according to the Diagnostic and Statistical Manual of Mental Disorders, 4th Edition (DSM-IV)) [3]. Since children with ADHD are unable to sustain attention on tasks, they may have greater difficulties with learning and academic success, their social lives and professional achievement [4]. In general, the ADHD_I_ and ADHD_C_ subtypes are clinically distinguishable in terms of cognitive deficits, inattention symptoms and behavioral problems. However, the neural mechanism underlying the pathophysiology of ADHD_C_ and ADHD_I_ is still not well understood.

Having a high temporal resolution (milliseconds), EEG can provide important information about the neural dynamics underlying functional network dysfunction in ADHD. Resting-state EEG studies have reported characteristic alterations in the spectral power of low- and high-frequency oscillations in ADHD children [5,6]. EEG source analysis has also shown age- and subtype-related modifications in cortical activity in ADHD children compared with typically developing individuals in different frequency bands [5].

An increasing body of evidence suggests that neural abnormalities are highly associated with atypical functional connectivity between different brain structures in ADHD patients [7,8,9,10,11,12,13,14,15]. Previous resting-state EEG studies have reported heterogeneous findings concerning alterations in brain connectivity in low- and high-frequency oscillations in different cortical regions in ADHD patients in the sensor space [9,16,17,18,19,20]. Liu et al. [21] reported significantly higher synchronization in high-frequency (alpha and beta) bands in ADHD children compared with their healthy controls. In contrast, Barry et al. [22] found elevated intrahemispheric coherence between the short-distance electrodes in the theta band, with reduced lateral differences in both the theta and alpha bands. Murias et al. [17] also reported significant differences in coherence between ADHD children and their controls in different frequency bands.

In the past decade, graph theory-based functional connectivity analysis has been used to characterize brain connectivity organization underpinning developmental disorders, including ADHD [9,16,23]. Few studies have reported atypical functional connectivity through graph analysis in ADHD patients [9,21,23,24,25]. Using the nonlinear fuzzy synchronization likelihood, Ahmadlou et al. [24] found characteristic alterations in the graph metrics in the left hemisphere of the brain in ADHD patients in the delta band. A reduction in global efficiency was observed in several studies [9,21,25], also reporting characteristic differences in other graph metrics between children with inattentive and combined ADHD and healthy individuals.

In recent years, there has been growing interest in identifying the densely connected cortical hubs forming rich clubs, which play a central role in global neural integration and brain communication through short pathways [26,27,28]. Since the presence of rich clubs in structural and functional brain networks ensures the functional efficiency of the brain networks, any damage to them might lead to brain diseases such as schizophrenia, migraines, dementia and ADHD [29,30,31,32,33]. To date, all of the EEG connectivity studies in ADHD have been performed in the sensor space, mostly using connectivity measures producing inflated connectivity estimates caused by volume conduction artifacts [34,35]. Moreover, to the best of our knowledge, no network study has been performed to investigate alterations in the rich-club organization of the brain using resting-state EEG in ADHD children. There has been only one study that explored the structural and functional rich-club organization of the brain using resting-state fMRI and diffusion tensor imaging in children with ADHD and autism spectrum disorders [33].

In this study, we aimed to investigate alterations in the rich-club organization of the brain networks in the source space in children with the most common subtypes of ADHD (ADHD_I_ and ADHD_C_) in comparison with typically developing individuals (TD). We examined two hypotheses: (1) patient groups would show frequency-specific alterations in functional connectivity, network topologies and rich-club organization in comparison with TD individuals, and (2) alterations in the resting-state regional source activity reported in ADHD children [5] would be associated with atypical functional connectivity. To test these hypotheses, the EEG source analysis was first performed on resting-state, high-density EEG data by the exact Low Resolution Electric Tomography software (eLORETA) in different frequency bands. The lagged phase synchronization (LPS), a nonlinear connectivity measure, was then used to construct brain networks. The graph theoretical analysis was then performed to investigate group differences in the topological properties and rich-club organization of functional networks between the ADHD and TD groups.

## 2. Materials and Methods

### 2.1. Participants

A total of 74 healthy controls (13 ± 2.3 years; 62 males and 12 females) and 67 ADHD children (12.9 ± 2.4 years; 55 males and 12 females) aged between 8 and 16 years were included in this study. The ethical approval was obtained from the local ethics committee at the SWPS University of Social Sciences and Humanities (approval No. 9/2016) and the Medical University of Warsaw (approval No. KB/157/2010). Informed consent was obtained from each child’s parent or caregiver according to the Declaration of Helsinki. The ADHD children were diagnosed based on the diagnostic criteria of the DSM-IV TR [3] and classified into inattentive (*n* = 20) and combined (*n* = 47) subtypes at the Public Pediatric Teaching Hospital in Warsaw, Poland [36]. The intensity of the ADHD symptoms was rated using the ADHD Rating Scale (ADHD-RS) [37] in terms of the total score (36.8 ± 10.9), inattention (20.3 ± 3.8), hyperactivity (9 ± 4.9) and impulsivity (8.5 ± 4.1) subscales. The participants had no previous head injuries causing loss of consciousness. The patients were asked not to take any medications at least 24 h before EEG recording. For the healthy controls, the parents were asked to fill out questionnaires regarding children’s health conditions to select participants with no attentional problems, neurological disorders or close family members with ADHD or ADD diagnoses.

### 2.2. Data Acquisition and Preprocessing

**Figure 1:**
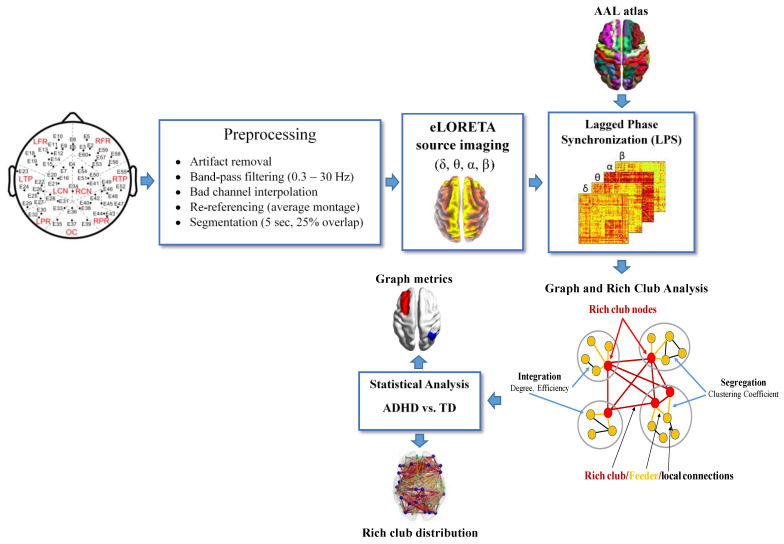
Processing pipeline, including preprocessing, source analysis, connectivity and graph analyses using high-density EEG in the source space.

As part of a larger study, five-minute resting-state EEG data were recorded from each participant using an EGI recoding system (Eugen, OR, USA) with 64 channels, a sampling frequency of 250 Hz and a referential montage referenced to the Cz electrode. The subjects were asked to remain still during data collection. The EEG recordings included three one-minute eyes-closed EEG epochs interleaved with periods of one-minute eyes-open intervals to prevent participants from falling asleep. The EEG data were first re-referenced using an average montage and preprocessed using the processing pipeline shown in Figure 1 [5]. In brief, the data preprocessing included artifact rejection, bad or noisy channel interpolation, band-pass filtering within 0.5–30 Hz and segmentation into five-second epochs with 25% overlap to reduce the data loss due to windowing [5], all of which was performed using custom-written routines in MATLAB (MathWorks, Natick, MA, USA), EEGlab (v2019.0, [38]) and Fieldtrip toolbox (v2019) [39]. Artifactual EEG segments, including eye movement and blinking, muscular activation and movement artifacts, were visually identified by an EEG expert and excluded from further analysis. The noisy or bad channels were replaced by interpolating their neighboring electrode data using the Fieldtrip toolbox. The average number of interpolated electrodes was 3 (±2.6) per subject [5].

### 2.3. EEG Source Analysis

Due to the insufficient length of the artifact-free eyes-open EEG data, we only performed EEG source analysis on the eyes-closed EEG epochs. To increase the statistical power, we only included participants who had a minimum of twenty artifact-free EEG segments for the connectivity analysis. Based on this criterion, 41 healthy controls (13 ± 2.3 years; 31 males and 10 females; see Figure S1 for age distribution) and 40 (10 inattentive and 30 combined) ADHD children (12.9 ± 2.4 years; 32 males and 8 females) were selected for connectivity analysis. From each subject, twenty artifact-free epochs were randomly selected to perform EEG source and connectivity analyses using the LORETA-KEY software package (www.uzh.ch/keyinst/LORETA.html (accessed on 18 July 2019)). To compute the cortical source density of the resting-state EEG data in the frequency domain, we used eLORETA [40], a discrete, linear 3D-weighted minimum norm inverse solution method shown to be robust in the presence of measurements and structured biological noise [5]. The cross-spectral matrices of the artifact-free EEG epochs were first computed to estimate the current source densities (CSDs) (intensity of the current or area, measured in A/m^2^) for each voxel in four frequency bands—delta (0.5–4 Hz), theta (4.25–8 Hz), alpha (8.25–13 Hz) and beta (13.25–30 Hz)—using the electrode positions provided by EGI [41]. The Montreal Neurologic Institute average MRI head model (MNI152) was used to restrict the source space within the gray matter, including 6239 voxels with a 5-mm spatial resolution [42]. We performed subject-wise normalization in different frequency bands by dividing the current source density value of every single voxel by the total activity of all voxels [5,43,44]. We further computed the average eLORETA solutions for 80 regions of interest (ROIs) defined according to the AAL atlas at the source level. The names, abbreviations and MNI coordinates of the ROIs can be found in Table S1.

### 2.4. Functional Connectivity Analysis

The lagged phase synchronization was calculated between all pairs of ROIs for each subject in different frequency bands. In comparison with functional connectivity methods relying on zero-lag connectivity like phase-locking value, LPS was shown to be resistant to non-physiological and volume conduction artifacts [35,45,46,47].

In each frequency band, a group-averaged connectivity matrix was computed for each group. The binarized matrices were then computed at the single-subject and group levels using optimal proportional thresholds (PTs) determined within a range of PTs from 5% to 35% with steps of 2.25%, based on the maximum global cost efficiency of the brain networks [48]. The optimal threshold (herein PT = 20%) was used to reduce the number of false-positive edges and minimize the noise [49].

The topological properties of the functional networks were quantified from the group binary connectivity matrices by computing the graph metrics, including the small-worldness, degree (*k*), global and local efficiency (GE and LE) and clustering coefficients (CC), as implemented in the Brain Connectivity Toolbox [50].

### 2.5. Rich-Club Organization

To investigate the rich-club organization of the group networks, for each degree *k* varying from 1 to the maximum degree in each network, a rich-club coefficient *φ*(*k*) was computed as follows:

(1) $$\phi (k)=\frac{2E_{>k}}{N_{>k}(N_{>k}-1)}$$

where *E*_>*k*_ and *N*_≥*k*_ (after removing all nodes with a degree less than *k*) represent the actual number of connections and the total number of possible connections between the remaining nodes if they are fully connected, respectively. The existence of rich clubs in the network was investigated by computing the ratio of the rich club coefficient as

(2) $$\phi_{norm}(k)=\frac{\phi (k)}{\phi_{rand}(k)}$$

where *ϕ_norm_*(*k*) is the normalized rich-club coefficient and *ϕ_rand_*(*k*) represents the average rich-club coefficient over a series of random networks of equal size with similar connectivity distributions, generated by randomizing the connections of the network while keeping the degree distribution of the matrix intact. For a given *k*, if *ϕ_norm_*(*k*) was more than one, nodes with a degree higher than *k* were considered rich-club nodes [29,51]. In our study, 1000 random networks were generated for each group average matrix.

In general, the choice of the *k* level is arbitrary and study-specific [26,52]. To compare the rich-club spatial distribution of the brain networks computed for the control and patient groups, we selected the *k* level in a way that had 40% of each network’s nodes ranked as rich clubs. In each network, connections were also classified into rich-club connections linking rich-club nodes, feeder connections linking rich-club to non-rich-club nodes and local connections linking non-rich-club nodes [26,53].

### 2.6. Statistical Analysis

We assessed the statistical significance of rich-club organization using permutation testing [54]. To this end, a null distribution of rich-club coefficients was computed using the distribution of *ϕ_rand_*(*k*), computed over 1000 random networks. The rich-club zones were defined as a range of k, in which *ϕ* significantly exceeded *ϕ_rand_*, with the *p*-value computed as the proportion of *ϕ_rand_* exceeding *ϕ*. Values of *p* < 0.05 were considered to indicate statistical significance. To assess differences in lagged phase synchronization between groups (ADHD_I_ vs. TD and ADHD_C_ vs. TD), the AAL regions were further grouped into ten brain regions—left and right frontal, central, temporal, parietal and occipital regions—listed in Table S1 based on the MNI coordinates at the source level. To assess the group differences in the network topology and LPS, non-parametric permutation *t*-tests with 1000 repetitions were used with *p* < 0.05 [48,55]. We also assessed the group differences at the nodal level. The results were then projected onto a 3D surface using BrainNet [56].

## 3. Results

### 3.1. Alterations in Functional Connectivity

**Figure 2:**
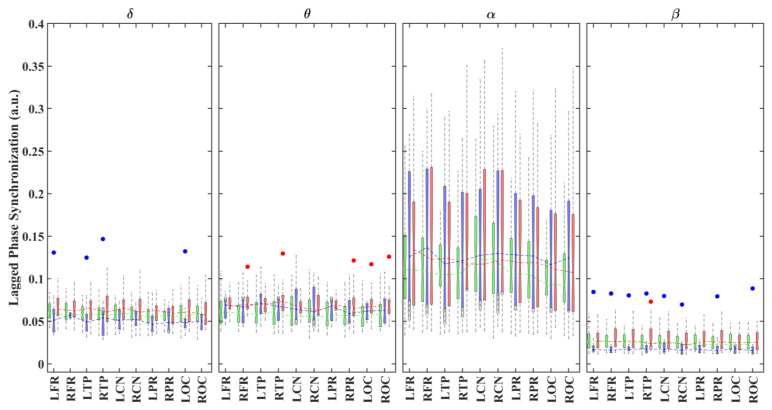
Boxplots displaying the median and interquartile ranges of the average (global) LPS values for each brain region and group (TD: green; ADHD_I_: blue; ADHD_C_: red) in different frequency bands. The points indicate statistically significant differences (*p* < 0.05, 1000 permutations) between the ADHD_C_ and TD (red) groups and between the ADHD_I_ and TD (blue) groups. R/L: right/left; FR: frontal regions; TP: temporal regions; CN: central regions; PR: parietal regions; OC: occipital regions.

Figure 2 shows boxplots for the average (global) LPS values for each brain region and group in different frequency bands. Overall, all groups showed slightly lower LPS values in the posterior areas in all frequency bands. Across all groups, the global LPS strengths were significantly stronger in α compared with the other frequency bands.

**Figure 3:**
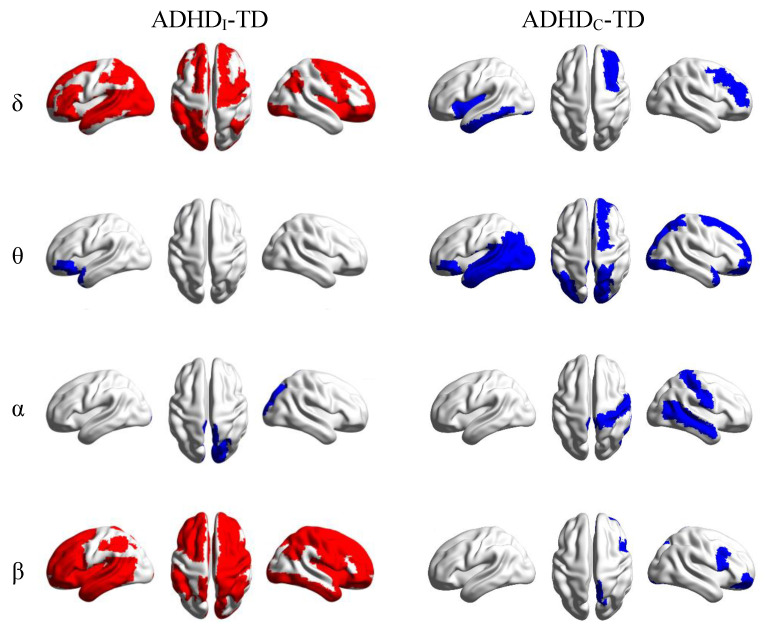
Statistical t-maps of significant differences (*p* < 0.05) in the average LPS strengths between the ADHD and TD groups in different frequency bands. Blue and red colors show significant increases and decreases in the average LPS in the ADHD_I_ and ADHD_C_ groups compared with TD individuals, respectively.

Figure 3 shows the t-maps of the significant differences (*p* < 0.05) in the global LPS strengths between the ADHD and TD groups in different frequency bands. Relative to the TD individuals, the ADHD_I_ group was characterized by a widespread significant decrease (*p* < 0.05) of the global LPS strength in the delta and beta bands. In the theta and alpha bands, this group showed significantly increased global LPS values in the left frontal and right occipital cortices, respectively. The statistical analysis also revealed significant increases in the global LPS strengths in the left temporal and posterior areas and the right frontal regions in the delta and theta bands, as well as the right temporal, central and frontal regions at higher frequencies (alpha and beta) in the ADHD_C_ group compared with the TD individuals.

### 3.2. Rich-Club Organization

The group functional networks exhibited a rich-club organization within the rich-club zones selected at a range of *k* levels within 4 ≤ *k* ≤ 28, 2 ≤ *k* ≤ 29, and 3 ≤ *k* ≤ 29 for the ADHD_I_, ADHD_C_ and TD groups, respectively. To explore alterations in the rich-club organization in ADHD patients, we chose a *k* level of 16 to have 40% of each functional network’s nodes ranked as rich clubs across different frequency bands.

**Figure 4:**
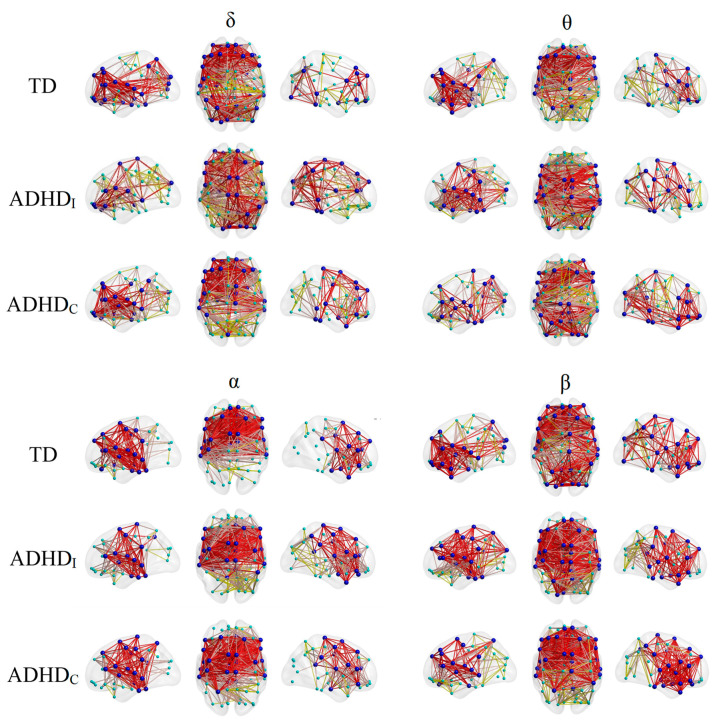
Lateral and axial views of the rich-club node (blue) distributions and connections (red edges) for each group in each frequency band. The feeder and local connections are shown in orange and yellow, respectively. Local nodes are shown in green. Nodes represent the centroids of the AAL regions.

Figure 4 illustrates the organization of the rich-club nodes and connections for each group in different frequency bands. For the TD individuals, the majority of the rich-club regions and connections were located in the frontal regions and posterior areas in the δ and β bands and the frontal and temporal or central regions in the θ and α bands. Relative to the TD group, the ADHD_I_ group was characterized by a reduction and an increase in the number of frontal and central rich clubs, respectively, in all frequency bands. The ADHD_I_ group also displayed lower numbers of δ and β rich clubs and a higher number of θ rich clubs in the posterior areas.

In both δ and β, the ADHD_C_ group showed a decrease and an increase in the number of rich-club regions and connections in the posterior areas and the right frontocentral regions, respectively, in comparison with the TD group. In θ, however, the posterior areas were highly involved in the rich-club organization of the brain in the ADHD_C_ group, relative to the TD group. In α, all groups presented similar rich-cub distributions, with a slight reduction in the number of frontal rich clubs in the ADHD_I_ group.

**Figure 5:**
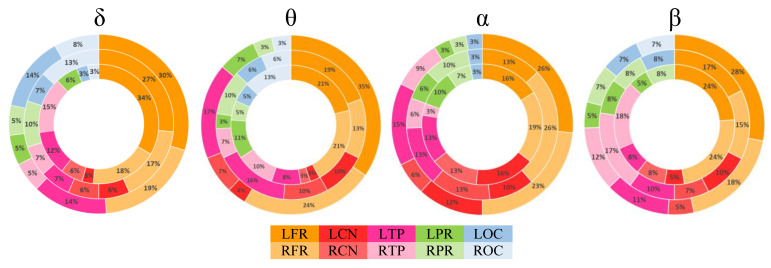
Regional proportion (percentage) of rich-club regions for the TD (outer ring), ADHD_I_ (middle ring), and ADHD_C_ (inner ring) groups in each frequency band.

**Figure 6:**
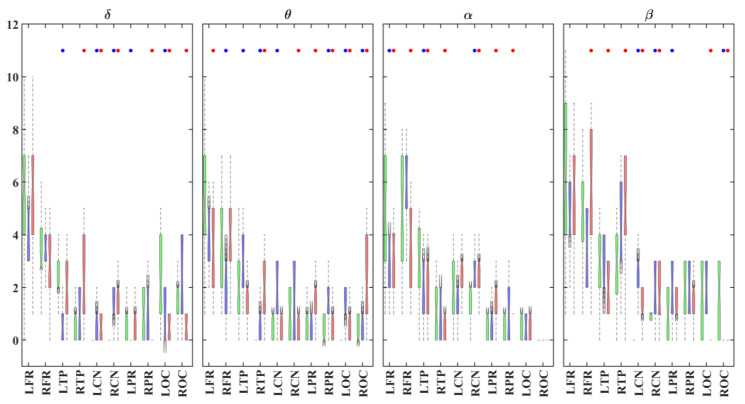
Boxplots displaying the median and interquartile ranges of the number of rich clubs for each brain region and group (TD: green; ADHD_I_: blue; ADHD_C_: red) in different frequency bands. The points indicate statistically significant differences (*p* < 0.05, 1000 permutations) between the ADHD_C_ and TD (red) groups and between the ADHD_I_ and TD (blue) groups. R/L: right/left; FR: frontal regions; TP: temporal regions; CN: central regions; PR: parietal regions; OC: occipital regions.

The rich-club proportions (RCP, expressed as a percentage) and rich-club numbers are shown in Figure 5 and Figure 6, respectively, for each brain region and group in different frequency bands. In all frequency bands and groups, most of the rich-club nodes were distributed in the bilateral frontal regions (Figure 5). Both ADHD groups displayed lower rich-club proportions in the frontal and temporal regions in comparison with the TD individuals (Figure 6). The bilateral temporal lobes with significant left laterality also showed high RCPs for all groups across all frequency bands, with the exception of the beta band. Regardless of the frequency band and group, the central and posterior areas showed lower RCPs. In the lower frequency bands (δ and θ), the bilateral temporal regions were more involved in the rich-club organization in the ADHD_C_ group, compared with the TD group (Figure 6). The right temporal lobe also showed higher RCPs in the δ and β bands in the ADHD_C_ group.

**Figure 7:**
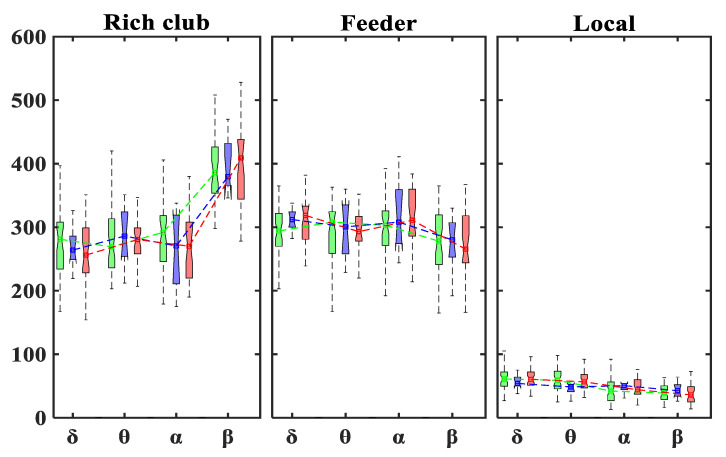
Boxplots displaying the median and interquartile ranges of the number of rich-club connections for each group (TD: green; ADHD_I_: blue; ADHD_C_: red) in different frequency bands.

As is shown in Figure 7, no significant differences were observed in the number of rich-club, feeder or local connections between the ADHD and TD groups in different frequency bands. All groups displayed a higher and lower number of rich-club and feeder connections, respectively, in the β band when compared with the other frequency bands. The number of local connections was significantly lower in comparison with those of the rich-club and feeder connections in all groups and frequency bands. A significant reduction in local connections was also observed in the α and β bands compared with the lower frequencies in all groups.

### 3.3. Alteration in Global and Local Topological Properties

**Figure 8:**
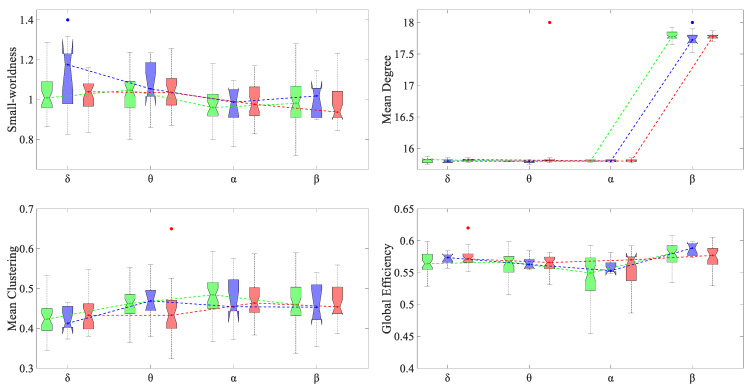
Average global graph measures for each group (TD: green; ADHD_I_: blue; ADHD_C_: red) in different frequency bands. The blue and red points indicate statistically significant differences (*p* < 0.05, non-parametric permutation) between the ADHD_I_ and TD groups and between the ADHD_C_ and TD groups, respectively.

Figure 8 shows the average graph measures for each group in different frequency bands at the network level. Regardless of the frequency band, all three groups showed small-worldness greater than one. In δ, the ADHD_I_ group displayed higher small-worldness in comparison with the TD group.

The network degree showed significant increases in the β band for all the groups. Compared with the TD group, both the ADHD_C_ and ADHD_I_ groups showed significantly increased and decreased network degrees in the θ and β bands, respectively. The ADHD_C_ group was further characterized by lower θ clustering coefficients and higher δ global efficiency relative to the TD group.

**Figure 9:**
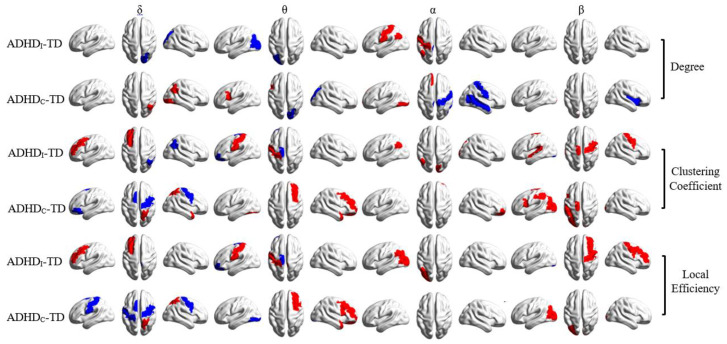
Statistical t-maps of significant differences (*p* < 0.05) in the nodal degree, nodal clustering coefficient and local efficiency between the ADHD and TD groups. Blue and red colors show significant increases and decreases in local graph metrics (degree, clustering coefficient and local efficiency) in ADHD_I_ or ADHD_C_ compared to TD, respectively.

Figure 9 illustrates the spatial distribution of differences in the nodal degree, nodal clustering coefficient and local efficiency between the ADHD and TD groups. Compared with the TD individuals, the ADHD_I_ group showed (1) significantly higher *k*, CC and LE values in the right posterior areas in the δ band, (2) a higher *k* value in the left occipital region and lower CC and LE values in the left central areas in the θ band, (3) lower *k*, CC and LE values in the left posterior areas in the α band and (4) a lower CC value in the bilateral central and left temporal areas and a lower LE value in the right frontocentral areas in the β band.

Relative to the TD group, the ADHD_C_ group displayed (1) significantly lower *k*, CC and LE values in the right posterior areas and higher CC and LE values in the bilateral central regions in the δ band, (2) a higher *k* value in the right occipital region and lower CC and LE values in the right frontal areas in the θ band, (3) a higher *k* value in the right central areas and lower *k*, CC and LE values in the frontal and occipital regions in the α band and (4) a higher *k* value in the right temporal region and lower CC and LE values in the left central and occipital regions in the β band.

**Figure 10:**
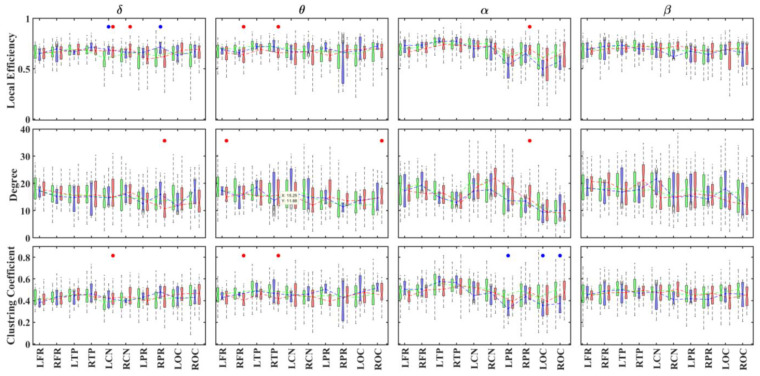
Boxplots displaying the median and interquartile ranges of the local degree, clustering coefficient and efficiency for each brain region and group (TD: green; ADHD_I_: blue; ADHD_C_: red) in different frequency bands. The points indicate statistically significant differences (*p* < 0.05, 1000 permutations) between the ADHD_C_ and TD groups (red) and between the ADHD_I_ and TD groups (blue). R/L: right/left; FR: frontal regions; TP: temporal regions; CN: central regions; PR: parietal regions; OC: occipital regions.

Similar trends of changes were observed for the graph metrics in the brain regions listed in Table S1 (Figure 10). However, in some cases, differences between the ADHD groups and the TD individuals were not significant because each brain region included several AAL regions, but significant differences were only limited to individual AAL regions.

## 4. Discussion

In our previous study [5], we investigated differences in the spectral power and current source densities (CSDs) between ADHD children and typically developing individuals using high-density resting-state EEG data. To investigate whether alterations in the resting-state EEG were due to regional changes in the CSDs or caused by changes in functional connectivity between brain regions, in this study, we used eLORETA and LPS to assess group differences in EEG source connectivity between the ADHD and TD children. We further investigated alterations in the rich-club organization of the brain networks in ADHD patients. Our results showed stronger alpha and weaker beta resting-state LPS values between different brain regions in comparison with the lower-frequency oscillations (delta and theta) in both the ADHD and TD groups. Compared with the TD group, the ADHD_I_ group was characterized by a widespread decrease in global LPS strengths in the delta and beta bands and an increase in the left frontal and right occipital regions in the theta and alpha bands, respectively. The ADHD_C_ group, however, displayed significant increases in its global LPS values, mostly observed in the central, temporal and posterior areas in different frequency bands. The majority of the rich-club nodes were distributed in the bilateral frontal regions, with significant leftward lateralization observed in all three groups. The main differences in the rich-club proportions were observed in the central and posterior areas between the ADHD and TD groups in a frequency-dependent manner. In all groups, significant differences in the network metrics, including the degree, clustering coefficient and efficiency, were observed between the low- (δ and θ) and high- (α and β) frequency oscillations. Compared with the TD group, the ADHD_I_ group showed a tendency for higher and lower degrees in the low- and high-frequency bands, respectively, in comparison with the TD individuals. The ADHD_C_ group showed an inverse trend. Both subtypes displayed lower regional clustering coefficients across different frequency bands relative to the TD group. Overall, our results supported both hypotheses in a frequency-specific manner. In what follows, we discuss the general implications of our results in each frequency band.

**Delta band:** As an indicator of maturational lags, alterations in the delta spectral powers (sensor space) and current source densities have been reported in ADHD patients compared with controls in the frontal, central or posterior areas [5,57,58,59,60,61,62,63,64]. In our study, we found higher delta rich-club proportions in the central areas in the ADHD groups compared with the TD group. The rich-club reorganization in the central regions might explain abnormalities in the delta spectral and source power reported in ADHD [5]. We also found significant alterations in the delta LPS in the right or bilateral frontal areas in both ADHD subtypes. Our results also showed significant increases in the delta LPS in the ADHD_C_ group, which is in line with the elevated frontal interhemispheric coherence reported in ADHD patients in the sensor space [65]. The disruption in the frontal FC might be the main reason for the alterations observed in the delta spectral power in both ADHD groups when compared with the TD group [5]. The ADHD_I_ group also showed a widespread decrease in global LPS strengths, in line with the reduced delta coherence reported in ADHD patients [66]. In addition to the synchronization level, we found significant differences in the rich-club distribution and proportion in the temporal, central and posterior areas between the ADHD and TD groups.

In the delta band, we found increased global efficiency for the ADHD_C_ group compared with the TD group. This finding is in agreement with the greater global efficiency reported in ADHD patients in the sensor space [8]. Relative to the TD group, both the ADHD_I_ and ADHD_C_ groups displayed alterations in the local network measures (*k*, CC and LE) as measures of the degree of functional integration and segregation of the brain networks. Our results are partly consistent with the findings reported in [24], which found leftward laterality in the average clustering coefficients and shortest path lengths in ADHD children. In the ADHD_I_ group, we found a leftward decrease in degree in the frontal region and a rightward increase in both the degree and clustering coefficient in the occipital regions. The ADHD_C_ group was also characterized by characteristic changes in the local network metrics, including decreases in the degree, CC and LE in the right parietal and occipital regions and a significant increase in the CC and LE in the bilateral central regions in comparison with the TD group.

**Theta band:** The enhanced theta activity in typically developing children is suggested to reflect information coding [67,68] and memory and attention processing [69,70,71]. In children with ADHD, increases in the theta power are suggested to be an indicator of drowsiness or “cortical slowing”, causing inattentiveness or hyperactivity in many resting-state EEG studies [5,22,58,59,60,62,72,73,74]. In our study, the ADHD_C_ group showed significant increases in the global LPS strengths in the right frontal and left temporal regions and also in the bilateral posterior areas. The ADHD_I_ group also showed increased LPS in the left frontal regions. These results are in line with the enhanced intrahemispheric frontal, temporal, central and posterior coherence observed in the theta band in ADHD children, particularly in ADHD_C_ children in different studies [22,75,76].

We also found lower and higher rich-club proportions in the frontal and temporoposterior regions, respectively, in both ADHD subtypes relative to the TD individuals. The disruption in the FC might be related to alterations in the theta spectral and source power observed in ADHD children in these regions [5,58]. In addition, the ADHD_C_ group showed significantly increased network degrees (functional integration), supporting the increases in the theta source power observed in ADHD children [5]. The ADHD_C_ group was further characterized by declined clustering coefficients relative to the TD group at the network level. The ADHD_I_ group also showed local alterations (mostly reduction) in the nodal clustering coefficients and local efficiencies in the frontal, central and occipital regions in the theta band. These observations reflect a reduction in the segregation of the brain networks in ADHD children in the source space. Our results are inconsistent with the results reported in other studies, suggesting greater average clustering coefficients in ADHD patients in the sensor space [8,9].

**Alpha band:** In healthy subjects, changes in the alpha power are suggested to be associated with cortical inhibition or excitation and cognitive or sensorimotor actions [77,78,79,80,81,82]. Compared with healthy children, the majority of the EEG studies performed on ADHD children have reported reduced alpha activity, especially in the posterior regions [58,61,83,84,85,86,87]. In ADHD patients, the attenuated alpha activity is suggested to be associated with a state of cortical hyperactivation, with increased excitability of the sensory cortices causing attentional self-control problems [77,78,86,87]. The reduced alpha power may also reflect deficits in processing visuospatial information and failure in sustained attention in ADHD children [74].

In our previous study, we found a significant widespread decrease in the alpha source power for ADHD_C_ children compared with TD individuals [5]. The global decrease in alpha activity has also been reported in adults with ADHD_C_ [88]. The elevated alpha activity observed in ADHD children in the sensor space [61,62,76,89,90] was also found in the source space in the prefrontal and posterior areas in ADHD_I_ children relative to TD individuals [5].

Few studies have investigated alterations in the alpha FC in ADHD patients in the sensor space [91,92,93]. In our study, both the ADHD and TD groups showed stronger average LPS values in the alpha band with similar spatial rich-club distributions. Both ADHD subtypes were characterized by significant increases in LPS in the right temporal, central or frontal lobes in comparison with the TD individuals. This result is consistent with the findings in other studies [91] reporting a rightward alpha asymmetry in the frontal and central regions in ADHD children compared with the healthy controls. We also observed lower local degrees, CC or LE in the frontal and occipital regions in both ADHD groups compared with the controls. Moreover, the ADHD_C_ group exhibited higher k values in the right central areas. These results are in contrast with those reported in [9], which found an increase in the average CC and LE in both ADHD subtypes in the sensor space.

**Beta band:** In healthy individuals, the increased beta activity is suggested to be associated with increased attention during physical and mental activities [94,95]. In children and adults with ADHD, significant increases in the resting-state theta activity have often been found to be accompanied by a significant decrease in beta power [18,60,62,73,92,96,97]. The alteration in the beta power in the frontal regions is suggested to be associated with poor inhibitory control and hyperactivity in ADHD children [98,99].

In our previous study, we found a significant diffuse decrease (*p* < 0.05) in the beta source power in ADHD_C_ children compared with TD individuals, in line with the findings in the sensor space [5,76,89]. In the ADHD_I_ children, however, we found significant increases in the beta source power in the frontal regions [5]. In the present study, we found significant increases in the number of beta rich-club connections in comparison with other frequency bands in ADHD and TD children. Relative to the TD children, the ADHD_I_ group was characterized by a widespread significant decrease (*p* < 0.05) in global LPS strengths and lower beta network degrees in the beta band in comparison with the TD group. The ADHD_C_ group also presented a trend for higher LPS strengths in the temporal, central and parietal regions. Both ADHD subtypes also showed lower rich-club proportions in the posterior areas accompanied by lower nodal CC and LE values observed in the frontal, central, left temporal and occipital regions. This finding is in agreement with the observations of Sidlauskaite et al. [100], who reported significantly lower CC values in the left temporal, occipital and frontal regions in the ADHD group. Our results suggest lower degrees of segregation for the functional networks in the beta band in ADHD children in comparison with TD individuals.

### Technical Considerations and Limitations

We found some discrepancies between our connectivity results found in the source space and those reported in the literature in the sensor space using low-density EEG data and measures based on zero-lag interactions [8,9,76,89,101]. In general, scalp EEG signals are the result of a linear mixture of source activities from all brain regions due to the volume conduction effect, which can lead to spurious correlations between the signals of short-distance electrodes [85,102]. To solve these problems, we performed source connectivity analysis using eLORETA to better characterize the alterations in functional connectivity patterns at the cortical level in ADHD children [103]. Since source connectivity analysis might also be affected by the source leakage [104], we further used lagged phase synchronization, which is shown to be resistant to non-physiological artifacts, volume conduction and low spatial resolution, which can significantly affect connectivity measures [102,105].

Finally, the main limitation of our study is the low sample size of the ADHD inattentive subgroup in comparison with the other two groups. The low sample size might bias the results due to low statistical power. We also did not investigate the effect of age on functional connectivity in ADHD children due to the sample size. In typically developing children, a decreasing and increasing trend with age has been found in the EEG power and current source density in low-frequency (delta and theta) and high-frequency (alpha and beta) bands, respectively [5]. Children with ADHD, however, exhibit increases and decreases in low and high-frequency power, respectively, with atypical trends of changes with age, especially in the frontal, temporal and central regions [5]. In ADHD children, the frequency-specific abnormality of the resting-state EEG power is often associated with hyperactivity and deficits in memory and attention processing, cortical inhibition or excitation and inhibitory control [5]. The regional alterations in the source power can be due to disrupted functional connectivity caused by ADHD-specific unbalanced interactions between local cortical networks and long-range corticocortical or subcortical activities [86]. Further investigation should be carried out to investigate the effect of age on the topological properties and rich-club organization of the brain networks in ADHD children. These issues require further data collection and analysis.

## 5. Conclusions

In this study, we used eLORETA and LPS to assess alterations in functional connectivity in ADHD children compared to healthy controls in different frequency bands using high-density EEG data under resting-state, eyes-closed conditions. We further investigated alterations in the rich-club organization of the brain networks in ADHD children. Our results showed significant alterations in the functional connectivity and rich-club distribution in the frontal, central and posterior areas in ADHD patients in a frequency-specific manner. Regardless of the frequency band, both ADHD groups showed higher and lower levels of functional integration in the θ and β bands, respectively. Both subtypes also displayed a reduced level of functional segregation relative to the TD individuals across different frequency bands. Our findings suggest that resting-state EEG source connectivity analysis is an efficient tool to better characterize the frequency-specific functional rich-club reorganization of the brain networks in association with the cognitive and attention deficits and symptomatology in ADHD children.

## Data Availability

Restrictions apply to the availability of these data. The data analyzed during the current study are available from Katarzyna Kuc and Anita Cybulska-Klosowicz on reasonable request. The data are not publicly available due to data protection and ethical concerns.

## Supplementary Materials

The following are available online at https://www.mdpi.com/article/10.3390/brainsci11070938/s1, Figure S1: Age distribution for ADHD and TD groups, Table S1: Correspondence between the brain regions shown in Figure 1 and AAL regions.

## References

[1] Bálint S., Czobor P., Komlósi S., Meszaros A., Simon V., Bitter I. (2009). Attention deficit hyperactivity disorder (ADHD): Gender-and age-related differences in neurocognition. Psychol. Med..

[2] Ayano G., Yohannes K., Abraha M. (2020). Epidemiology of attention-deficit/hyperactivity disorder (ADHD) in children and adolescents in Africa: A systematic review and meta-analysis. Ann. Gen. Psychiatry.

[3] (2000). Text Revision.

[4] Nigg J.T. (2001). Is ADHD a disinhibitory disorder?. Psychol. Bull..

[5] Ahmadi M., Kazemi K., Kuc K., Cybulska-Klosowicz A., Zakrzewska M., Racicka-Pawlukiewicz E., Helfroush M.S., Aarabi A. (2020). Cortical source analysis of resting state EEG data in children with attention deficit hyperactivity disorder. Clin. Neurophysiol..

[6] Newson J.J., Thiagarajan T.C. (2019). EEG frequency bands in psychiatric disorders: A review of resting state studies. Front. Hum. Neurosci..

[7] Furlong S. (2018). Resting-State EEG Connectivity in Young Children with ADHD: A Potential Neural Marker. Ph.D. Thesis.

[8] Michelini G., Jurgiel J., Bakolis I., Cheung C.H., Asherson P., Loo S.K., Kuntsi J., Mohammad-Rezazadeh I. (2019). Atypical functional connectivity in adolescents and adults with persistent and remitted ADHD during a cognitive control task. Transl. Psychiatry.

[9] Ghaderi A.H., Nazari M.A., Shahrokhi H., Darooneh A.H. (2017). Functional brain connectivity differences between different ADHD presentations: Impaired functional segregation in ADHD-combined presentation but not in ADHD-inattentive presentation. Basic Clin. Neurosci..

[10] Fair D.A., Posner J., Nagel B.J., Bathula D., Dias T.G.C., Mills K.L., Blythe M.S., Giwa A., Schmitt C.F., Nigg J.T. (2010). Atypical default network connectivity in youth with attention-deficit/hyperactivity disorder. Biol. Psychiatry.

[11] Tomasi D., Volkow N.D. (2012). Abnormal functional connectivity in children with attention-deficit/hyperactivity disorder. Biol. Psychiatry.

[12] Tian L., Jiang T., Wang Y., Zang Y., He Y., Liang M., Sui M., Cao Q., Hu S., Peng M. (2006). Altered resting-state functional connectivity patterns of anterior cingulate cortex in adolescents with attention deficit hyperactivity disorder. Neurosci. Lett..

[13] Peterson B.S., Potenza M.N., Wang Z., Zhu H., Martin A., Marsh R., Plessen K.J., Yu S. (2009). An FMRI study of the effects of psychostimulants on default-mode processing during Stroop task performance in youths with ADHD. Am. J. Psychiatry.

[14] Cubillo A., Halari R., Ecker C., Giampietro V., Taylor E., Rubia K. (2010). Reduced activation and inter-regional functional connectivity of fronto-striatal networks in adults with childhood Attention-Deficit Hyperactivity Disorder (ADHD) and persisting symptoms during tasks of motor inhibition and cognitive switching. J. Psychiatr. Res..

[15] Monk C.S., Peltier S.J., Wiggins J.L., Weng S.-J., Carrasco M., Risi S., Lord C. (2009). Abnormalities of intrinsic functional connectivity in autism spectrum disorders. Neuroimage.

[16] Wang Y., Tao F., Zuo C., Kanji M., Hu M., Wang D. (2019). Disrupted Resting Frontal–Parietal Attention Network Topology is Associated with a Clinical Measure in Children with Attention-Deficit/Hyperactivity Disorder. Front. Psychiatry.

[17] Murias M., Webb S.J., Greenson J., Dawson G. (2007). Resting state cortical connectivity reflected in EEG coherence in individuals with autism. Biol. Psychiatry.

[18] Clarke A.R., Barry R.J., McCarthy R., Selikowitz M., Johnstone S.J., Hsu C.-I., Magee C.A., Lawrence C.A., Croft R.J. (2007). Coherence in children with attention-deficit/hyperactivity disorder and excess beta activity in their EEG. Clin. Neurophysiol..

[19] Dupuy F.E., Clarke A.R., Barry R.J., McCarthy R., Selikowitz M. (2008). EEG coherence in girls with attention-deficit/hyperactivity disorder: Stimulant effects in good responders. Int. J. Psychophysiol..

[20] Barry R.J., Clarke A.R., McCarthy R., Selikowitz M., Johnstone S.J. (2005). EEG coherence adjusted for inter-electrode distance in children with attention-deficit/hyperactivity disorder. Int. J. Psychophysiol..

[21] Liu T., Chen Y., Lin P., Wang J. (2015). Small-world brain functional networks in children with attention-deficit/hyperactivity disorder revealed by EEG synchrony. Clin. EEG Neurosci..

[22] Barry R.J., Clarke A.R., McCarthy R., Selikowitz M. (2002). EEG coherence in attention-deficit/hyperactivity disorder: A comparative study of two DSM-IV types. Clin. Neurophysiol..

[23] Wang B., Wang G., Wang X., Cao R., Xiang J., Yan T., Li H., Yoshimura S., Toichi M., Zhao S. (2021). Rich-club analysis in adults with ADHD connectomes reveals an abnormal structural core network. J. Atten. Disord..

[24] Ahmadlou M., Adeli H., Adeli A. (2012). Graph theoretical analysis of organization of functional brain networks in ADHD. Clin. EEG Neurosci..

[25] Cao M., Shu N., Cao Q., Wang Y., He Y. (2014). Imaging functional and structural brain connectomics in attention-deficit/hyperactivity disorder. Mol. Neurobiol..

[26] Van den Heuvel M.P., Sporns O. (2013). An anatomical substrate for integration among functional networks in human cortex. J. Neurosci..

[27] Colizza V., Flammini A., Serrano M.A., Vespignani A. (2006). Detecting rich-club ordering in complex networks. Nat. Phys..

[28] McAuley J.J., da Fontoura Costa L., Caetano T.S. (2007). Rich-club phenomenon across complex network hierarchies. Appl. Phys. Lett..

[29] de Reus M.A., van den Heuvel M.P. (2013). Rich club organization and intermodule communication in the cat connectome. J. Neurosci..

[30] Collin G., de Nijs J., Pol H.H., Cahn W., van den Heuvel M.P. (2016). Connectome organization is related to longitudinal changes in general functioning, symptoms and IQ in chronic schizophrenia. Schizophr. Res..

[31] Li K., Liu L., Yin Q., Dun W., Xu X., Liu J., Zhang M. (2017). Abnormal rich club organization and impaired correlation between structural and functional connectivity in migraine sufferers. Brain Imaging Behav..

[32] Daianu M., Mezher A., Mendez M.F., Jahanshad N., Jimenez E.E., Thompson P.M. (2016). Disrupted rich club network in behavioral variant frontotemporal dementia and early-onset A lzheimer’s disease. Hum. Brain Mapp..

[33] Ray S., Miller M., Karalunas S., Robertson C., Grayson D.S., Cary R.P., Hawkey E., Painter J.G., Kriz D., Fombonne E. (2014). Structural and functional connectivity of the human brain in autism spectrum disorders and attention-deficit/hyperactivity disorder: A rich club-organization study. Hum. Brain Mapp..

[34] Nunez P.L., Srinivasan R., Westdorp A.F., Wijesinghe R.S., Tucker D.M., Silberstein R.B., Cadusch P.J. (1997). EEG coherency: I: Statistics, reference electrode, volume conduction, Laplacians, cortical imaging, and interpretation at multiple scales. Electroencephalogr. Clin. Neurophysiol..

[35] Nolte G., Bai O., Wheaton L., Mari Z., Vorbach S., Hallett M. (2004). Identifying true brain interaction from EEG data using the imaginary part of coherency. Clin. Neurophysiol..

[36] Giertuga K., Zakrzewska M.Z., Bielecki M., Racicka-Pawlukiewicz E., Kossut M., Cybulska-Klosowicz A. (2017). Age-related changes in resting-state EEG activity in attention deficit/hyperactivity disorder: A cross-sectional study. Front. Hum. Neurosci..

[37] DuPaul G.J., Power T.J., Anastopoulos A.D., Reid R. (1998). ADHD Rating Scale—IV: Checklists, Norms, and Clinical Interpretation.

[38] Delorme A., Makeig S. (2004). EEGLAB: An open source toolbox for analysis of single-trial EEG dynamics including independent component analysis. J. Neurosci. Methods.

[39] Oostenveld R., Fries P., Maris E., Schoffelen J.-M. (2011). FieldTrip: Open source software for advanced analysis of MEG, EEG, and invasive electrophysiological data. Comput. Intell. Neurosci..

[40] Pascual-Marqui R.D. (2002). Standardized low-resolution brain electromagnetic tomography (sLORETA): Technical details. Methods Find. Exp. Clin. Pharm..

[41] Luu P., Ferree T. (2005). Determination of the HydroCel Geodesic Sensor Nets’ Average Electrode Positions and Their 10–10 International Equivalents.

[42] Mazziotta J., Toga A., Evans A., Fox P., Lancaster J., Zilles K., Woods R., Paus T., Simpson G., Pike B. (2001). A probabilistic atlas and reference system for the human brain: International Consortium for Brain Mapping (ICBM). Philos. Trans. R. Soc. Lond. Ser. B Biol. Sci..

[43] Zinn M., Zinn M., Valencia I., Jason L., Montoya J. (2018). Cortical hypoactivation during resting EEG suggests central nervous system pathology in patients with Chronic Fatigue Syndrome. Biol. Psychol..

[44] Paquette V., Beauregard M., Beaulieu-Prévost D. (2009). Effect of a psychoneurotherapy on brain electromagnetic tomography in individuals with major depressive disorder. Psychiatry Res. Neuroimaging.

[45] Rizkallah J., Amoud H., Fraschini M., Wendling F., Hassan M. (2020). Exploring the correlation between M/EEG source–space and fMRI networks at rest. Brain Topogr..

[46] Stam C.J., Jones B., Nolte G., Breakspear M., Scheltens P. (2007). Small-world networks and functional connectivity in Alzheimer’s disease. Cereb. Cortex.

[47] Canuet L., Ishii R., Pascual-Marqui R.D., Iwase M., Kurimoto R., Aoki Y., Ikeda S., Takahashi H., Nakahachi T., Takeda M. (2011). Resting-state EEG source localization and functional connectivity in schizophrenia-like psychosis of epilepsy. PLoS ONE.

[48] Bassett D.S., Bullmore E.T., Meyer-Lindenberg A., Apud J.A., Weinberger D.R., Coppola R. (2009). Cognitive fitness of cost-efficient brain functional networks. Proc. Natl. Acad. Sci. USA.

[49] Drakesmith M., Caeyenberghs K., Dutt A., Lewis G., David A., Jones D.K. (2015). Overcoming the effects of false positives and threshold bias in graph theoretical analyses of neuroimaging data. Neuroimage.

[50] Rubinov M., Sporns O. (2010). Complex network measures of brain connectivity: Uses and interpretations. Neuroimage.

[51] Khalilian M., Kazemi K., Fouladivanda M., Makki M., Helfroush M.S., Aarabi A. (2021). Effect of Multishell Diffusion MRI Acquisition Strategy and Parcellation Scale on Rich-Club Organization of Human Brain Structural Networks. Diagnostics.

[52] Harriger L., Van Den Heuvel M.P., Sporns O. (2012). Rich club organization of macaque cerebral cortex and its role in network communication. PLoS ONE.

[53] Fouladivanda M., Kazemi K., Makki M., Khalilian M., Danyali H., Gervain J., Aarabi A. (2021). Multi-scale structural rich-club organization of the brain in full-term newborns: A combined DWI and fMRI study. J. Neural Eng..

[54] Van Den Heuvel M.P., Sporns O., Collin G., Scheewe T., Mandl R.C., Cahn W., Goñi J., Pol H.E.H., Kahn R.S. (2013). Abnormal rich club organization and functional brain dynamics in schizophrenia. JAMA Psychiatry.

[55] Bernhardt B.C., Chen Z., He Y., Evans A.C., Bernasconi N. (2011). Graph-theoretical analysis reveals disrupted small-world organization of cortical thickness correlation networks in temporal lobe epilepsy. Cereb. Cortex.

[56] Xia M., Wang J., He Y. (2013). BrainNet Viewer: A network visualization tool for human brain connectomics. PLoS ONE.

[57] Clarke A.R., Barry R.J., McCarthy R., Selikowitz M. (1998). EEG analysis in attention-deficit/hyperactivity disorder: A comparative study of two subtypes. Psychiatry Res..

[58] Clarke A.R., Barry R.J., McCarthy R., Selikowitz M. (2001). Age and sex effects in the EEG: Differences in two subtypes of attention-deficit/hyperactivity disorder. Clin. Neurophysiol..

[59] Clarke A.R., Barry R.J., McCarthy R., Selikowitz M. (2001). Age and sex effects in the EEG: Development of the normal child. Clin. Neurophysiol..

[60] Bresnahan S.M., Anderson J.W., Barry R.J. (1999). Age-related changes in quantitative EEG in attention-deficit/hyperactivity disorder. Biol. Psychiatry.

[61] Bresnahan S.M., Barry R.J. (2002). Specificity of quantitative EEG analysis in adults with attention deficit hyperactivity disorder. Psychiatry Res..

[62] Koehler S., Lauer P., Schreppel T., Jacob C., Heine M., Boreatti-Hümmer A., Fallgatter A., Herrmann M. (2009). Increased EEG power density in alpha and theta bands in adult ADHD patients. J. Neural Transm..

[63] Kamida A., Shimabayashi K., Oguri M., Takamori T., Ueda N., Koyanagi Y., Sannomiya N., Nagira H., Ikunishi S., Hattori Y. (2016). EEG power spectrum analysis in children with ADHD. Yonago Acta Med..

[64] Shephard E., Tye C., Ashwood K.L., Azadi B., Asherson P., Bolton P.F., McLoughlin G. (2018). Resting-state neurophysiological activity patterns in young people with ASD, ADHD, and ASD+ ADHD. J. Autism Dev. Disord..

[65] Greber M., Klein C., Leipold S., Sele S., Jäncke L. (2020). Heterogeneity of EEG resting-state brain networks in absolute pitch. Int. J. Psychophysiol..

[66] Murias M., Swanson J.M., Srinivasan R. (2007). Functional connectivity of frontal cortex in healthy and ADHD children reflected in EEG coherence. Cereb. Cortex.

[67] Klimesch W. (1999). EEG alpha and theta oscillations reflect cognitive and memory performance: A review and analysis. Brain Res. Rev..

[68] Kahana M.J., Seelig D., Madsen J.R. (2001). Theta returns. Curr. Opin. Neurobiol..

[69] Michels L., Bucher K., Lüchinger R., Klaver P., Martin E., Jeanmonod D., Brandeis D. (2010). Simultaneous EEG-fMRI during a working memory task: Modulations in low and high frequency bands. PLoS ONE.

[70] Başar E., Schürmann M., Sakowitz O. (2001). The selectively distributed theta system: Functions. Int. J. Psychophysiol..

[71] Dietl T., Dirlich G., Vogl L., Lechner C., Strian F. (1999). Orienting response and frontal midline theta activity: A somatosensory spectral perturbation study. Clin. Neurophysiol..

[72] Clarke A.R., Barry R.J., Heaven P.C., McCarthy R., Selikowitz M., Byrne M.K. (2008). EEG in adults with attention-deficit/hyperactivity disorder. Int. J. Psychophysiol..

[73] Kitsune G.L., Cheung C.H., Brandeis D., Banaschewski T., Asherson P., McLoughlin G., Kuntsi J. (2015). A matter of time: The influence of recording context on EEG spectral power in adolescents and young adults with ADHD. Brain Topogr..

[74] Saha P., Mukhopdhyay P., Chakraborty P., Poria S., Mukundan C., Sharma S., Ghosh P., Vijay M., Nath S., Ghosh S. (2017). Neural oscillations in resting state EEG in ADHD children-A preliminary study. J. Indian Assoc. Child Adolesc. Ment. Health.

[75] Montagu J. (1975). The hyperkinetic child: A behavioural, electrodermal and EEG investigation. Dev. Med. Child Neurol..

[76] Chabot R.J., Serfontein G. (1996). Quantitative electroencephalographic profiles of children with attention deficit disorder. Biol. Psychiatry.

[77] Romei V., Brodbeck V., Michel C., Amedi A., Pascual-Leone A., Thut G. (2007). Spontaneous fluctuations in posterior α-band EEG activity reflect variability in excitability of human visual areas. Cereb. Cortex.

[78] Haegens S., Händel B.F., Jensen O. (2011). Top-down controlled alpha band activity in somatosensory areas determines behavioral performance in a discrimination task. J. Neurosci..

[79] Mathewson K.E., Lleras A., Beck D.M., Fabiani M., Ro T., Gratton G. (2011). Pulsed out of awareness: EEG alpha oscillations represent a pulsed-inhibition of ongoing cortical processing. Front. Psychol..

[80] Klimesch W., Sauseng P., Hanslmayr S. (2007). EEG alpha oscillations: The inhibition–timing hypothesis. Brain Res. Rev..

[81] Klimesch W. (2012). Alpha-band oscillations, attention, and controlled access to stored information. Trends Cogn. Sci..

[82] Smith M.E., McEvoy L.K., Gevins A. (1999). Neurophysiological indices of strategy development and skill acquisition. Cogn. Brain Res..

[83] Lazzaro I., Gordon E., Whitmont S., Plahn M., Li W., Clarke S., Dosen A., Meares R. (1998). Quantified EEG activity in adolescent attention deficit hyperactivity disorder. Clin. Electroencephalogr..

[84] Loo S.K., Hale T.S., Macion J., Hanada G., McGough J.J., McCracken J.T., Smalley S.L. (2009). Cortical activity patterns in ADHD during arousal, activation and sustained attention. Neuropsychologia.

[85] Loo S.K., Makeig S. (2012). Clinical utility of EEG in attention-deficit/hyperactivity disorder: A research update. Neurotherapeutics.

[86] Woltering S., Jung J., Liu Z., Tannock R. (2012). Resting state EEG oscillatory power differences in ADHD college students and their peers. Behav. Brain Funct..

[87] Deiber M.-P., Hasler R., Colin J., Dayer A., Aubry J.-M., Baggio S., Perroud N., Ros T. (2019). Linking alpha oscillations, attention and inhibitory control in adult ADHD with EEG neurofeedback. Neuroimage Clin..

[88] Loo S.K., Hale T.S., Hanada G., Macion J., Shrestha A., McGough J.J., McCracken J.T., Nelson S., Smalley S.L. (2010). Familial clustering and DRD4 effects on electroencephalogram measures in multiplex families with attention deficit/hyperactivity disorder. J. Am. Acad. Child Adolesc. Psychiatry.

[89] Clarke A.R., Barry R.J., Dupuy F.E., Heckel L.D., McCarthy R., Selikowitz M., Johnstone S.J. (2011). Behavioural differences between EEG-defined subgroups of children with attention-deficit/hyperactivity disorder. Clin. Neurophysiol..

[90] Poil S.-S., Bollmann S., Ghisleni C., O’Gorman R., Klaver P., Ball J., Eich-Höchli D., Brandeis D., Michels L. (2014). Age dependent electroencephalographic changes in attention-deficit/hyperactivity disorder (ADHD). Clin. Neurophysiol..

[91] Hale T.S., Smalley S.L., Dang J., Hanada G., Macion J., McCracken J.T., McGough J.J., Loo S.K. (2010). ADHD familial loading and abnormal EEG alpha asymmetry in children with ADHD. J. Psychiatr. Res..

[92] Lenartowicz A., Mazaheri A., Jensen O., Loo S.K. (2018). Aberrant modulation of brain oscillatory activity and attentional impairment in attention-deficit/hyperactivity disorder. Biol. Psychiatry Cogn. Neurosci. Neuroimaging.

[93] Nazari M.A., Wallois F., Aarabi A., Berquin P. (2011). Dynamic changes in quantitative electroencephalogram during continuous performance test in children with attention-deficit/hyperactivity disorder. Int. J. Psychophysiol..

[94] Ackerman P.T., Dykman R.A., Oglesby D.M., Newton J.E. (1994). EEG power spectra of children with dyslexia, slow learners, and normally reading children with ADD during verbal processing. J. Learn. Disabil..

[95] Andreassi J. (1995). Psychophysiology: Human Behavior and Physiological Response.

[96] Buyck I., Wiersema J.R. (2014). Resting electroencephalogram in attention deficit hyperactivity disorder: Developmental course and diagnostic value. Psychiatry Res..

[97] Tye C., McLoughlin G., Kuntsi J., Asherson P. (2011). Electrophysiological markers of genetic risk for attention deficit hyperactivity disorder. Expert Rev. Mol. Med..

[98] Clarke A.R., Barry R.J., McCarthy R., Selikowitz M. (2001). Excess beta activity in children with attention-deficit/hyperactivity disorder: An atypical electrophysiological group. Psychiatry Res..

[99] Clarke A.R., Barry R.J., McCarthy R., Selikowitz M. (2001). EEG-defined subtypes of children with attention-deficit/hyperactivity disorder. Clin. Neurophysiol..

[100] Sidlauskaite J., Sonuga-Barke E., Roeyers H., Wiersema J.R. (2016). Altered intrinsic organisation of brain networks implicated in attentional processes in adult attention-deficit/hyperactivity disorder: A resting-state study of attention, default mode and salience network connectivity. Eur. Arch. Psychiatry Clin. Neurosci..

[101] Michelini G., Jurgiel J., Bakolis I., Cheung C.H., Asherson P., Loo S.K., Kuntsi J., Mohammad-Rezazadeh I. (2018). Atypical functional connectivity in adolescents and adults with persistent and remitted ADHD. bioRxiv.

[102] Stam C.J., Nolte G., Daffertshofer A. (2007). Phase lag index: Assessment of functional connectivity from multi channel EEG and MEG with diminished bias from common sources. Hum. Brain Mapp..

[103] Pascual-Marqui R.D., Michel C.M., Lehmann D. (1994). Low resolution electromagnetic tomography: A new method for localizing electrical activity in the brain. Int. J. Psychophysiol..

[104] Schoffelen J.M., Gross J. (2009). Source connectivity analysis with MEG and EEG. Hum. Brain Mapp..

[105] Pascual-Marqui R.D., Lehmann D., Koukkou M., Kochi K., Anderer P., Saletu B., Tanaka H., Hirata K., John E.R., Prichep L. (2011). Assessing interactions in the brain with exact low-resolution electromagnetic tomography. Philos. Trans. R. Soc. A Math. Phys. Eng. Sci..

