# Supplementary material for: Disrupted Functional Rich-Club Organization of the Brain Networks in Children with Attention-Deficit/Hyperactivity Disorder, a Resting-State EEG Study
Source: Brain Sci. 2021 Jul 16;11(7):938. doi: 10.3390/brainsci11070938 (PMC8305540; doi:10.3390/brainsci11070938)
Supplement: Supplementary file 1 [file brainsci-11-00938-s001.zip › brainsci-1271542-supplementary.pdf]

Supplementary Materials

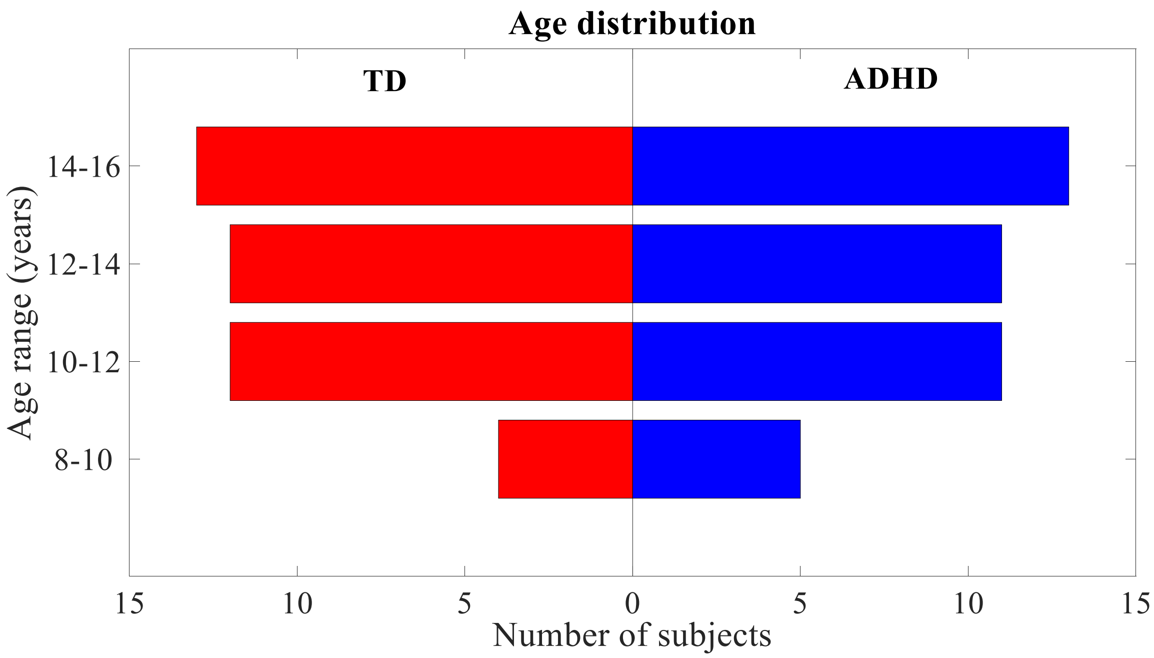

Figure S1. Age distribution for ADHD and TD groups.

Table S1. Correspondence between the brain regions shown in Figure 1 and AAL regions.

Brain region	AAL Region	Abbreviations	MNI coordinates		
			X	Y	Z
Left FR	Frontal_Sup_L	L-SFG	−18.450	34.810	42.200
	Frontal_Sup_Orb_L	L-SFGorb	−16.560	47.320	−13.310
	Frontal_Mid_L	L-MFG	−33.430	32.730	35.460
	Frontal_Mid_Orb_L	L-MFGorb	−30.650	50.430	−9.6200
	Frontal_Inf_Oper_L	L-IFGoper	−48.430	12.730	19.020
	Frontal_Inf_Tri_L	L-IFGtri	−45.580	29.910	13.990
	Frontal_Inf_Orb_L	L-IFGorb	−35.980	30.710	−12.110
	Rolandic_Oper_L	L-ROL	−47.160	−8.4800	13.950
	Olfactory_L	L-OLF	−8.0600	15.050	−11.460
	Frontal_Sup_Medial_L	L-SFGm	−4.8	49.17	30.89
	Frontal_Med_Orb_L	L-MedFGorb	−5.17	54.06	−7.4
	Rectus_L	L-REC	−5.08	37.07	−18.14
	Insula_L	L-INS	−35.13	6.65	3.44
	Cingulum_Ant_L	L-ACC	−4.04	35.4	13.95
Right FR	Frontal_Sup_R	R-SFG	21.900	31.120	43.820
	Frontal_Sup_Orb_R	R-SFGorb	18.490	48.100	−14.020
	Frontal_Mid_R	R-MFG	37.590	33.060	34.040
	Frontal_Mid_Orb_R	R-MFGorb	33.180	52.590	−10.730
	Frontal_Inf_Oper_R	R-IFGoper	50.200	14.980	21.410
	Frontal_Inf_Tri_R	R-IFGtri	50.330	30.160	14.170
	Frontal_Inf_Orb_R	R-IFGorb	41.220	32.230	−11.910
	Rolandic_Oper_R	R-ROL	52.650	−6.2500	14.630
	Olfactory_R	R-OLF	10.43	15.91	−11.26
	Frontal_Sup_Medial_R	R-SFGm	9.1	50.84	30.22
	Frontal_Med_Orb_R	R-MedFGorb	8.16	51.67	−7.13
	Rectus_R	R-REC	8.35	35.64	−18.04
	Insula_R	R-INS	39.02	6.25	2.08
	Cingulum_Ant_R	R-ACC	8.46	37.01	15.84

**Left CN**	Supp_Motor_Area_L	L-SMA	−5.3200	4.8500	61.380
	Precentral_L	L-PreCG	−38.650	−5.6800	50.940
	Postcentral_L	L-PoCG	−42.46	−22.63	48.92
	Paracentral_Lobule_L	L-PaCL	−7.63	−25.36	70.07
	Cingulum_Mid_L	L-DCC	−5.48	−14.92	41.57
**Right CN**	Supp_Motor_Area_R	R-SMA	8.6200	4.1700	61.850
	Precentral_R	R-PreCG	41.370	−8.2100	52.090
	Postcentral_R	R-PoCG	41.43	−25.49	52.55
	Paracentral_Lobule_R	R-PaCL	7.48	−25.59	68.09
	Cingulum_Mid_R	R-DCC	8.02	−8.83	39.79
**Left TP**	Temporal_Sup_L	L-STG	−53.16	−20.68	7.130
	Temporal_Pole_Sup_L	L-STGpol	−39.88	15.14	−20.18
	Temporal_Mid_L	L-MTG	−55.52	−33.80	−2.20
	Temporal_Pole_Mid_L	L-MTGpol	−36.32	14.59	−34.08
	Temporal_Inf_L	L-ITG	−49.77	−28.05	−23.17
	Hippocampus_L	L-HIP	−25.03	−20.74	−10.13
	ParaHippocampal_L	L-PHG	−21.17	−15.95	−20.70
	Amygdala_L	L-AMYG	−23.27	−0.67	−17.14
**Right TP**	Temporal_Sup_R	R-STG	58.15	−21.78	6.80
	Temporal_Pole_Sup_R	R-STGpol	48.25	14.75	−16.86
	Temporal_Mid_R	R-MTG	57.47	−37.23	−1.47
	Temporal_Pole_Mid_R	R-MTGpol	44.22	14.55	−32.23
	Temporal_Inf_R	R-ITG	53.69	−31.07	−22.32
	Hippocampus_R	R-HIP	29.23	−19.78	−10.33
	ParaHippocampal_R	R-PHG	25.38	−15.15	−20.47
	Amygdala_R	R-AMYG	27.32	0.64	−17.50
**Left PR**	Parietal_Sup_L	L-SPG	−23.45	−59.56	58.96
	Parietal_Inf_L	L-IPL	−42.80	−45.82	46.74
	SupraMarginal_L	L-SMG	−55.79	−33.64	30.45
	Precuneus_L	L-PCUN	−7.24	−56.07	48.01
	Angular_L	L-ANG	−44.14	−60.82	35.59
	Cingulum_Post_L	L-PCC	−4.85	−42.92	24.67
**Right PR**	Parietal_Sup_R	R-SPG	26.11	−59.18	62.06
	Parietal_Inf_R	R-IPL	46.46	−46.29	49.54
	SupraMarginal_R	R-SMG	57.61	−31.50	34.48
	Angular_R	R-ANG	45.51	−59.98	38.63
	Precuneus_R	R-PCUN	9.98	−56.05	43.77
	Cingulum_Post_R	R-PCC	7.44	−41.81	21.87
**Left OC**	Calcarine_L	L-CAL	−7.14	−78.67	6.44
	Cuneus_L	L-CUN	−5.93	−80.13	27.22
	Lingual_L	L-LING	−14.62	−67.56	−4.63
	Occipital_Sup_L	L-SOG	−16.54	−84.26	28.17
	Occipital_Mid_L	L-MOG	−32.39	−80.73	16.11
	Occipital_Inf_L	L-IOG	−36.36	−78.29	−7.84
	Fusiform_L	L-FFG	−31.16	−40.30	−20.23
**Right OC**	Calcarine_R	R-CAL	15.99	−73.15	9.40
	Cuneus_R	R-CUN	13.51	−79.36	28.23
	Lingual_R	R-LING	16.29	−66.93	−3.87
	Occipital_Sup_R	R-SOG	24.29	−80.85	30.59
	Occipital_Mid_R	R-MOG	37.39	−79.70	19.42
	Occipital_Inf_R	R-IOG	38.16	−81.99	−7.61
	Fusiform_R	R-FFG	33.97	−39.10	−20.18

FR: frontal regions; TP: temporal regions; CN: central regions; PR: parietal regions; OC: occipital regions.
